# Dyskinesia-hyperpyrexia syndrome in Parkinson's disease triggered by overdose of levodopa — a case report and literature review

**DOI:** 10.3389/fneur.2023.1323717

**Published:** 2024-01-05

**Authors:** Xiangnan Du, Xuemei Wang, Xiaokun Geng

**Affiliations:** ^1^Department of Neurology, Beijing Luhe Hospital, Capital Medical University, Beijing, China; ^2^China-America Institute of Neuroscience, Department of Neurology, Beijing Luhe Hospital, Capital Medical University, Beijing, China

**Keywords:** Parkinson's disease, acute hyperpyrexia syndrome, dyskinesia, hyperpyrexia, treatment

## Abstract

Dyskinesia-hyperpyrexia syndrome, a rare medical emergency in Parkinson's disease, is first described in 2010. It is characterized by severe continuous dyskinesia associated with rhabdomyolysis, hyperthermia and subsequent alteration of the mental state. Gradual reduction of dopaminergic dose or DBS is recommended treatment. The prognosis is usually good, but sometimes fatal. But so far, this potentially fatal complication is not widely recognized by clinicians. In emergency, if clinicians fail to make prompt diagnosis and treatment, patients' conditions may get worse, and their lives may be threatened in serious cases.

## Introduction

Acute hyperpyrexia syndrome related to Parkinson's disease usually occurs in advanced PD patients, especially in those who have received long-term treatment with levodopa. Acute hyperpyrexia syndrome includes Parkinson hyperpyrexia syndrome (PHS), dyskinesia-hyperpyrexia syndrome (DHS) and serotonin syndrome (SS) (1, 2). These acute hyperthermia syndromes are easily confused by clinicians. If emergency physicians cannot recognize these three hyperthermia syndromes timely and give treatment optimally, it may lead to poor prognosis, even threaten lives. Here, we reported a PD patient who presented with severe dyskinesia-hyperpyrexia syndrome, meanwhile performed a comprehensive literature.

## Case description

Our patient was a 78-year-old female with a history of PD for 10 years. She had been taking levodopa/benserazide 600/150 mg/day, pramipexole 0.75 mg/day and selegiline 10 mg/day. She denied a history of current statins or serotoninergic drugs use. The season was summer during which she had a slight weight loss. A week before admission she had head trauma secondary to a fall, followed by appetite loss accompanied with a mild dyskinesia. The patient's family considered the symptoms to be tremors and added another compound tablet of levodopa/benserazide (200/50 mg), along with daily oral medication, including levodopa/benserazide 600/150 mg, pramipexole 0.75 mg and selegiline 10 mg, the LEDD was 975 mg. After that the abnormal movements worsened significantly.

**Figure 1:**
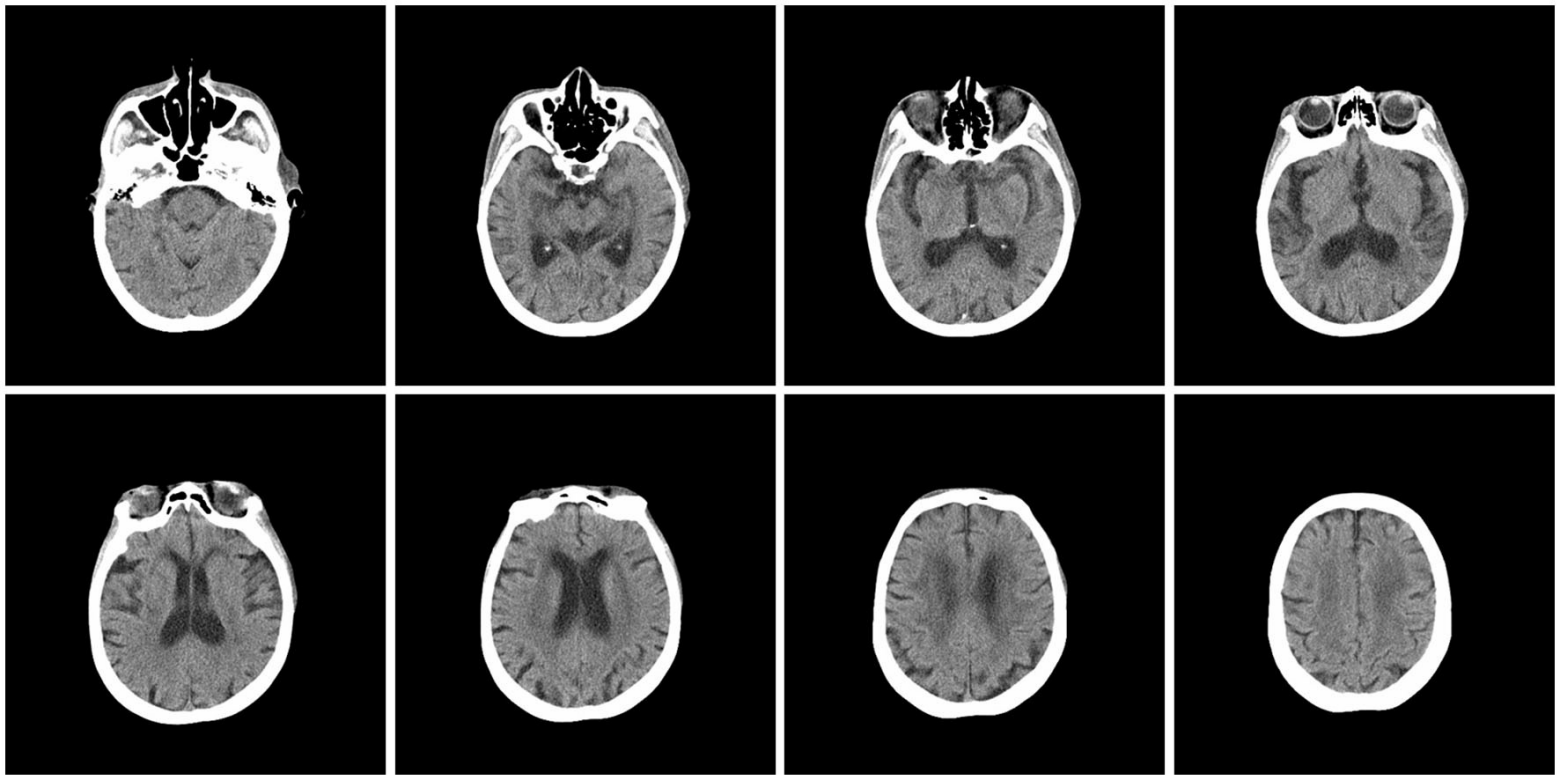
Head CT basically ruled out intracranial hemorrhage, head injury and strategic infarction.

On admission, she was in coma because of hypoglycemia, even though she had no history of diabetes and never received hypoglycemic treatment. In the emergency room, her body temperature was 39.6°C, and the heart rate was 118 beats/min. When hypoglycemia is rapidly corrected, the patient's consciousness recovered and severe generalized involuntary dyskinesia of limbs occurred, more intense in the upper limbs. Laboratory findings were mild leukocytosis (white blood cell count 10.07 × 10^9^/L). Elevated BUN (13.6 mmol/L) with serum creatinine (87umol/L) indicated a mild decrease in renal function. Lac (8.4 mmol/L) and serum CK level (1579 U/L) were highly elevated. A chest CT scan showed ill-defined ground-glass opacity in right lower lobes. No space-occupying inflammatory lesion was observed. Head CT basically ruled out intracranial hemorrhage, head injury and strategic infarction (Figure 1). Unfortunately, No head MRI was performed due to the patient's involuntary movements. Since low PCT (0.1 ng/ml) and bacterial culture of sputum were negative, pneumonia was not considered as a fever source, and antibiotics were not prescribed.

Based on the clinical features and laboratory findings, a diagnosis of dyskinesia hyperpyrexia syndrome was made. We discontinued pramipexole and reduced the levodopa/benserazide dose to 300/75 mg/day. The dose of antiparkinsonian drugs was reduced progressively but the dyskinesia never disappeared. After the patient's drug was completely reduced on the 10th day of hospitalization, the abnormal movement completely disappeared but the patient developed fever again. So we added selegiline 5 mg/day, and after that the patient did not show any abnormal movement, with the temperature returned to normal.

## Discussion

Acute hyperpyrexia syndrome related to Parkinson's disease is rare but life-threatening complication of PD. Approximately 30–40% of PD patients who have been treated with levodopa for more than 5 years may develop levodopa-induced dyskinesia (3, 4). When a PD patient experiences acute hyperpyrexia, the PHS, DHS and SS need to be considered. Wang's study elucidated the similarities and differences between PHS, DHS, and SS (5). The similarity of these three syndromes can exhibit hyperpyrexia, neuromuscular symptoms, autonomic symptoms and consciousness disturbance. Concerning potential triggers, it should be noted that high ambient temperature, dehydration, infection, and trauma can be common triggers for PHS and DHS, while excessive serotonin drugs are the only cause of SS. In terms of disease course, the course of PHS and DHS usually lasts for 1–2 weeks, while the course of SS usually lasts for < 24 h. The neuromuscular symptoms of DHS are often manifested as dyskinesia, while PHS is often characterized by rigidity and oligokinesia. Compare to PHS, SS may have symptoms such as increased tendon reflexes and clonus in addition to rigidity. PHS and SS usually manifest as tachycardia, sweating, and unstable blood pressure as autonomic nervous symptoms, which are rare in DHS. The consciousness disorder of PHS usually manifests as a decrease in consciousness level from drowsiness to coma, while DHS typically manifests as blurred consciousness and hallucinations. SS usually exhibit anxiety and irritability, while severe patients may experience symptoms such as delirium and coma. The most effective treatment for the three syndromes is to remove potential triggers. PHS needs to gradually increase the dosage of dopaminergic drugs or restart DBS. Conversely, DHS needs to gradually reduce the dosage of dopaminergic drugs or reduce the stimulation of DBS. In addition, SS needs to discontinue the serotonergic drugs. Dyskinesia associated with hyperpyrexia was first described in a 68-year-old advanced PD patient by Gil-Navarro and Grandas (6). And DHS is even rarer compared with PHS or SS. To date, a total of 15 cases of DHS have been reported in 12 publications (Table 1). Sometimes, the dyskinesia may lead to rhabdomyolysis, acute renal failure and respiratory distress, which may become severe and life-threatening.

**Table 1:** Overview of 14 affected patients with dyskinesia hyperpyrexia syndrome.

**References**	**Age/gender**	**PD duration**	**Suspected provocation factor**	**Season**	**Peak body T (°C)**	**Symptoms**	**LEDD (mg)**	**Treatment**	**Outcome**
Gil-Navarro and Grandas (6)	68/F	12	NA	NA	41.2	Generalized dyskinesias confusion and hallucination Tachycardia CK = 1455 IU/L	1680	Intravenous fluids antipyretic agents pramipexole tapered off Quetiapine 25mg	Recovered
Lyoo and Lee (7)	74/M	17	Dopaminergic drug dose increase (levodopa 1050 mg increased to 3400 mg)	NA	38.2	Generalized dyskinesias consciousness was normal acute renal injury (176.8 μmol/L) CK = 24651 IU/L	3400	Dopaminergic drug was stopped sedative	Recovered
Taguchi et al. (8)	70/F	13	Dopaminergic drug form change (pramipexole IR to ER)	Autumn	40.3	Generalized dyskinesias confusion and hallucination Tachycardia CK = 35000 IU/L	950	Intravenous fluids antipyretic agents reduced dopaminergic drugs	Recovered
Herreros-Rodriguez and Sanchez-Ferro (9)	74/F	16	High ambient temperature	Summer	40.2	Generalized dyskinesias Consciousness was normal CK = 178–2509 IU/L	1390	Receive an LCIG (1310 mg/d)	Died from an unrelated pulmonary embolism
Sánchez-Herrera et al. (10)	66/F	16	High ambient temperature, dopaminergic drug dose increase	Summer	40.2	Generalized dyskinesias confusion and hallucination CK = 7177 IU/L	1810	Intravenous fluids antipyretic agents sedative LCIG reduced amantadine, ropinirole and safinamide were stopped	Recovered
Baek et al. (11)	74/F	23	Trauma, infection	Spring	40.3	Generalized dyskinesias confusion and hallucination aspiration pneumonia acute renal injury (142μmol/L) CK = 10230 IU/L	675	LCIG reduced Amantadine and pramipexole were stopped Antibiotics Sedative	Recovered
Sarchioto et al. (12)	80/M	20	High ambient temperature, infection	Summer	42	Generalized dyskinesias confusion and lethargy Acute renal failure (186.5μmol/L) pneumonia CK = 16040 IU/L	1550	Pramipexole and AMA withdrawn LCIG dose reduced to 700mg antibiotics	Death
	76/F	18	High ambient temperature/infection	Summer	41	Generalized dyskinesias stupor pneumonia respiratory failure CK = 2967 IU/L	1060	Antibiotics	Death
	79/F	30	High ambient temperature/infection	Summer	39.5	Generalized dyskinesias consciousness was normal pneumonia acute renal injury (175μmol/L) CK = 1967 IU/L	1000	Intravenous fluids LCIG reduction (675 mg) antibiotics	Recovered
Novelli et al. (13)	62/M	34	High ambient temperature, infection	Summer	40.7	Generalized dyskinesias confusion tachycardia CK = 4891 IU/L	2528	Intravenous fluids antipyretic agents antibiotics DBS reduced to 1 V bilaterally Levodopa/carbidopa 125/12.5mg plus entacapone 200 mg for six daily doses	Recovered
Zu et al. (14)	76/F	16	Dopaminergic drug dose increase	NA	40.2	Generalized dyskinesias unconsciousness tachycardia CK = 2489 IU/L	1150	Reduced dopaminergic drugs	Recovered
Pitakpatapee et al. (15)	64/M	10	Delayed gastric emptying time	NA	37.5	Generalized dyskinesia consciousness was normal sweating CK = 4246 IU/L	967	Intravenous fluids stopped all medications sedative	Recovered
	61/F	10	Infection, increasing dose of ropinirole	NA	37.8	Generalized dyskinesia consciousness was normal dehydration myalgia CK = 12094 IU/L	1222	Rehydration stopped all medications sedative	Recovered
Wang et al. (16)	74/F	4	Dopaminergic drug dose increase	Autumn	39.7	Generalized dyskinesias confusion and hallucination tachycardia CK = 821 IU/L	1500	Rehydration stopped all medications sedative	Recovered
Luo et al. (17)	55/M	10	Dopaminergic drug dose increase	Summer	40.6	Generalized dyskinesias confusion tachycardia CK = 1468 IU/L	1750	Rehydration reduced dopaminergic drugs sedative	Recovered

NA, not available.

In our review, we found that DHS was more likely to appear in advanced PD patients accompanied with motor symptom fluctuation, particularly with high-dose dopaminergic therapy. The dyskinesia in DHS patients is usually systemic and persistent and may precede fever. Therefore, some patients may only present with dyskinesia without elevated body temperature in the early stages. The body temperature may fluctuate from 37.5°C to 42°C, and most of which is above 40°C. Elevations of CK are generally considered as secondary to severe dyskinesia, ranging from hundreds to 35000 IU/L, but not all DHS patients CK elevation. There were no characteristic changes through our patient's head CT scan. We speculate that other imaging examinations may have more implications. Previously study rarely described characteristic neuroimaging features of DHS. A recent research provides new insights into DHS and expands on its neuroimaging features. Luo's study showed that DHS can cause reversible encephalopathy, which was detected through head MRI (17). Thus, DHS still has many characteristics to be discovered. In previous study, there were 9 out of 14 patients manifested as confusion and hallucinations, which might be caused by dopaminergic hyperactivity in the mesocorticolimbic system. Three patients manifested with consciousness level reduction (stupor or lethargy). Furthermore, we observed that female patients are prone to develop this complication (ten female and four male). These gender differences might be induced by female hormonal patterns which could increase the individual dyskinetic sensitivity to levodopa (18). Besides, compared with male, female patients with lighter body weight are more likely to have higher plasma concentrations of levodopa with the same treatment protocol (19). Therefore, clinicians should pay more attention to individualized drug therapy.

The pathogenesis is still unclear. Previous literature review suggested a variety of suspected triggering factors, including adjustment in dopaminergic therapy, trauma, infection, hot weather, dehydration, gastrointestinal diseases and changes in deep brain stimulation (5). Dopamine dysregulation syndrome (DDS), an uncommon complication of medical treatment for PD, is characterized by addictive behavior and excessive use of dopaminergic medication. Levodopa is more likely to be associated with DDS compared to other drugs; in addition, there were higher rates of dyskinesias and motor fluctuations. Warren's study suggested that individuals at risk may have less efficient inhibitory dopaminergic systems. Thus, we speculate that DDS is also one of the reasons for DHS (20). The current hypothesis was considered as that DHS are prone to occur in high temperature environments. Dopamine is an important neurotransmitter in the hypothalamus, which can promote body heat dissipation and play an important role in regulating body temperature. In patients with advanced PD, degeneration of dopaminergic neurons in the substantia nigra leads to dopaminergic deficiency (10). Therefore, it is speculated that in summer, it is easier to trigger dyskinesia in advanced PD patients treated with high-dose dopaminergic drugs when under high ambient temperature and long daylight duration which may increase the dopaminergic activity (16). Furthermore, hyperpyrexia was also related to increased thermogenesis caused by excessive dyskinesia.

The combination of reduced dopaminergic medications with standard medical care is usually effective and the prognosis is generally favorable. Prompt identification and optimal treatment may improve patient outcomes. Reducing dopaminergic drug dosage as soon as possible is the most effective way to treat DHS. Sedation is effective for patients with refractory dyskinesia (15). Supportive treatments such as intravenous rehydration, cooling, anti-infection, and electrolyte balance are also critical for DHS. Among the 13 patients reported so far, only two patients died due to DHS (12). Complications including rhabdomyolysis, acute renal failure, and respiratory failure suggest a poor prognosis for DHS.

## Data availability statement

The datasets presented in this study can be found in online repositories. The names of the repository/repositories and accession number(s) can be found in the article/supplementary material.

## Ethics statement

Ethical review and approval was not required for the study on human participants in accordance with the local legislation and institutional requirements. Written informed consent from the patients/participants or patients/participants' legal guardian/next of kin was not required to participate in this study in accordance with the national legislation and the institutional requirements. Written informed consent was obtained from the individual(s) for the publication of any potentially identifiable images or data included in this article.

## Author contributions

XD: Writing – original draft, Writing – review & editing. XW: Writing – review & editing. XG: Supervision, Writing – review & editing.
